# Mechanical Characterization and Constitutive Modeling of Nano-Stabilized Soil under Uniaxial Compression

**DOI:** 10.3390/ma16041488

**Published:** 2023-02-10

**Authors:** Xingchen Zhang, Jianen Gao, Minmin Qiang, Haochen Zhang, Xinghua Li, Shaobo Long, Zhe Gao, Henghui Fan

**Affiliations:** 1 Institute of Soil and Water Conservation, Northwest Agriculture and Forestry University, Xianyang 712100, China; 2 Northwest Engineering Corporation Limited, Power China, Xi’an 710065, China; 3 College of Civil Engineering, Yan’an University, Yan’an 716000, China; 4 College of Water Resources and Architectural Engineering, Northwest Agriculture and Forestry University, Xianyang 712100, China

**Keywords:** constitutive model, stress–strain curve, unconfined compressive strength, nano-stabilized soil

## Abstract

The stress–strain constitutive model under uniaxial compression is a basic element and important characterization method for determining physical and mechanical properties in cement-based materials research. In this study, a stress–strain constitutive model under uniaxial compression was established, which was based on a new nano-stabilized soil (NSS) through typical mechanical tests and constitutive relationship research. The results indicate that the unconfined compressive strength (UCS) of the nano-stabilized soil was enhanced with the increase in curing period and nano-stabilizer dosage, and that the strength growth rate reaches the maximum at a 12% dosage in the tested samples. The UCS of NSS under a 12% dosage is about 10~15% higher than that of ordinary stabilized soil (SS) without nano doping, and 25~40% higher compared with grade 42.5 cement-soil. The established constitutive model could accurately describe the linear-elastic and elastic-plastic deformation characteristics of NSS under uniaxial compression, which will be conducive to revealing the curve variation law of the stress–strain process. The research results could provide scientific support for the theoretical innovation and engineering application of green environmental protection materials.

## 1. Introduction

As a new green material for the efficient utilization of soil and water resources, the soil nano-stabilizer was developed innovatively on the basis of traditional cement-based soil stabilizers [1,2,3]. It can not only improve the micro interface structure and macro mechanical properties of normal soil at room temperature [4] but also possesses the characteristics of high strength, strong durability and a simple preparation process. The above superiorities mean that the nano-stabilizer shows broad application potential in the Loess Plateau and other areas lacking rock and sand [5,6,7].

The research on soil stabilizers arose in the United States, Japan and other countries in the 1970s. In recent years, with the continuous application of soil stabilizers in the fields of architecture, transportation and environment, investigators have conducted various studies and achieved positive results regarding its hydration process, consolidation theory and mechanical properties. Mahedi compared the effectiveness of cement, lime and fly ashes in improving the engineering properties of expansive soils, and proved that 10~12% calcium oxide in stabilizers had the best stabilizing effect on expansive soils [8]. Ahmadi found that different specific surface areas of nano-silicon caused clay water absorption differences due to the different filling methods and particle encapsulation forms [9]. Yang used serial block-face scanning electron microscopy (SBFSEM) to study the three-dimensional morphological characteristics of cement hydration products, improving the observation methods and accuracy [10]. Along with the rapid development of nanomaterials and technology since the start of the 21st century, much research on nano-modified stabilizers and stabilized soil has been conducted, respectively, from the perspective of physical structure, chemical composition and interface characterization. Rostami studied the effect of the branched polymers containing nano-silica on the microstructure of cement slurry by means of TGA methods, and testified that the two materials can improve the fluidity of cement slurry [11]. Choobbasti studied the effect of nano-silica on the microstructure and mechanical properties of cemented sand using SEM and XRD, and obtained the optimal mixing dosage of nano-silica [12]. These positive results contributed to the theoretical exploration and technical breakthroughs related to cement-based stabilized soil. However, research is relatively weak on the variation regularities of microstructures and their quantitative response mechanism to macro mechanical properties [13,14]. In particular, the research on the stress–strain constitutive model of new cement-based materials represented by nano-stabilized soil is still limited.

As a mathematical characterization method to describe the macroscopic properties of materials, constitutive models of cement-based stabilized soil are the basis for exploring the mechanical properties [15,16,17]. Owing to the specific feature of various soil stabilizers, besides the diversity and random-ness of soil properties, there is no consensus on the construction of stress–strain constitutive models for typical soil stabilizers. Nano-stabilized soil is mainly used for subgrade, pavement and other foundation-bearing purposes in practical projects [18,19]. The compressive performance was often the focus of research in the field of engineering materials; nevertheless, the actual deformation characteristics are complicated in the face of anisotropic multiaxial compression [20]. In order to reveal the essence of the multiaxial compression deformation of nano-stabilized soil, the first step should be to expound the basic uniaxial compression stress–strain relationship. Nano-stabilized soil (NSS) is a cement-based composite material with certain strength and durability, which is formed by mixing and compacting the nano-stabilizer, soil and water at certain proportions [21]. Due to the obvious linear-elastic properties, the stress–strain characteristics of NSS are different from those of ordinary soil. Simultaneously, it also has a typical plastic deformation process that differs from concrete. In other words, the NSS stress–strain constitutive relationship should be somewhere between soil and concrete [17,22], and it would be more suitable for the deformation characteristics to regard NSS as an elastoplastic material. Therefore, the constitutive model of NSS under uniaxial compression could be investigated by referring to the stress–strain curve law of concrete [23,24].

Accordingly, in view of the shortcomings of the existing uniaxial constitutive model in characterizing the microscopic variation of nano-stabilized soil, this research was carried out in order to explore the constitutive relationship of nano-stabilized soil with elastoplastic characteristics on the basis of previous studies, so as to improve the theoretical construction of cement-based stabilized soil materials. In this study, the influence of different curing periods and dosages on the compressive strength of nano-stabilized soil was investigated by means of a laboratory unconfined compressive strength test [25], and a uniaxial compressive constitutive model suitable for nano-stabilized soil was constructed based on the piecewise stress–strain curve characteristics of concrete [26], which provided a reference for the investigation of the anisotropic multiaxial constitutive model. These research results could not only contribute to improving the theoretical construction of the deformation process of cement-based stabilized soil but also provide a scientific basis for the further investigation of new green materials such as nano-stabilized soil.

## 2. Materials and Methods

### 2.1. Nano-Stabilized Soil

Nano-stabilized soil is composed of nanoparticles, a stabilizer and soil in general. The physical and chemical properties of different nanoparticles and soil stabilizers vary widely, resulting in the diversity of microstructure and mechanical properties of stabilized soil. The nano-stabilized soil used in this study was prepared by mixing a soil nano-stabilizer (N-MBER, Nanometer Material Becoming Earth into Rock) into the soil collected from the Loess Plateau [27,28]. The N-MBER was mainly composed of nanoparticles, gelling agent, alkaline catalyst, retarder, surfactant and grinding aid. The type and content of the above ingredients are shown in Table 1.

**Figure 1:**
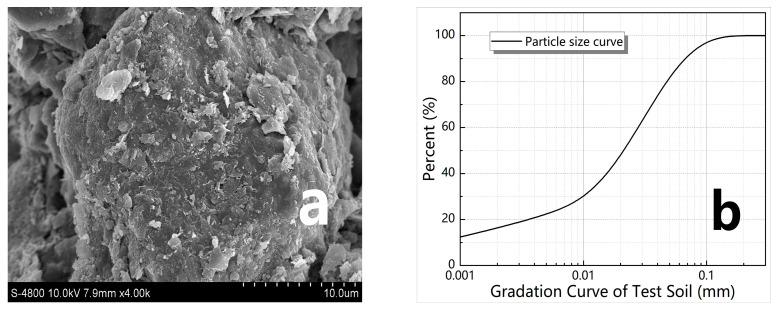
Soil structure and gradation. (**a**) SEM of the soil structure; (**b**) gradation curve of soil.

The test loess was obtained from Yan’an City, Shaanxi Province. The loess was found to be a low-liquid limit clay of CL with a specific gravity of 2.68, a plasticity index of 13.1, an optimal water content of 16.8%, a maximum dry density of 1.76 g/cm^3^, and a median particle size of 24.5 μm, among which the particles ranging from 5 μm to 75 μm in diameter accounted for about 67%. The chemistry of the soil was dominated by silicon oxide and alumina, which made up nearly 70% of the content. Figure 1a shows an image of the loess structure magnified 4000 times, which was taken by a type S-4800 field emission scanning electron microscope (SEM), made by Hitachi in Tokyo, Japan. It can be seen from Figure 1a that the interior of the loess was composed of soil microparticles which had different morphologies, and multistage pores with various forms, both of which were arranged and cemented together to form a typical porous aggregate. Figure 1b presents the gradation curve of test soil. It can be seen from Figure 1b that 65% of the loess used in the test is silty clay with particles between 0.005 and 0.075 mm in size.

### 2.2. Sample Preparation

The test samples were divided into three groups, which were nano-stabilized soil (NSS), ordinary stabilized soil (SS) and grade 42.5 cement soil (CM). The NSS was composed of N-MBER mixed with loess, and the dosage of N-MBER was designed to be 6%, 9%, 12%, 15% and 18% of the mass fraction of the mixture. The SS, as a control group, was a mixture of common stabilizer (MBER) and loess. The other control group was made of grade 42.5 cement mixed with loess. In order to simplify the test groups and facilitate comparison, the dosages of the stabilizers in the two control groups were both designed to be 12%. The preparation and curing of the samples were carried out by the methods specified in the ‘test methods of materials stabilized with inorganic binders for highway engineering (JTG E51-2009)’ [29]. The samples were pressed into cylindrical blocks with the size of Φ 50 mm × 50 mm by means of the bidirectional static pressing method to prepare for the strength test. In order to ensure the quality of the test and prevent possible accidental errors, 6 duplicate samples were prepared in each group. After preparation, the samples were placed under the condition of 20 ± 2 °C and relative humidity greater than 95% for curing. The designed curing periods of this experiment were 7 days, 28 days and 90 days, respectively. The mix proportions of each experimental group are shown in Table 2.

### 2.3. Uniaxial Compression Test

**Figure 2:**
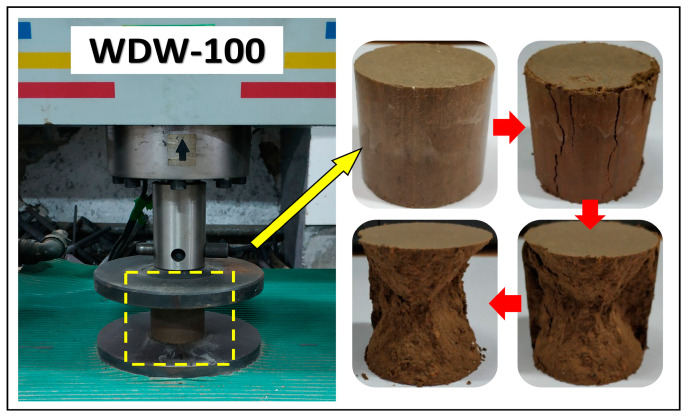
The unconfined compressive strength test (The red arrows represented the process of failure of the sample).

After curing for the specified period, the unconfined compressive strength (UCS) test was carried out on each group of samples. Before the test, the height and weight of the samples should be measured to compare the changes in volume and water absorption after curing. The UCS test was carried out using a WDW-100 microcomputer hydraulic universal testing machine, made by Changchun New Testing Machine CO., LTD, Changchun, China. In order to unify the test method and facilitate the strength comparison between test samples, a constant rate of 1 mm/min specified was used for loading. The stress–strain data were recorded by the software of the testing machine during the test. Eventually, the stress–strain constitutive relationship of NSS under uniaxial compression could be analyzed and predicted by using the UCS test data. The unconfined compressive strength test and the sample failure process are shown in Figure 2.

## 3. Results and Discussions

### 3.1. Strength Variation of Uniaxial Compression

**Figure 3:**
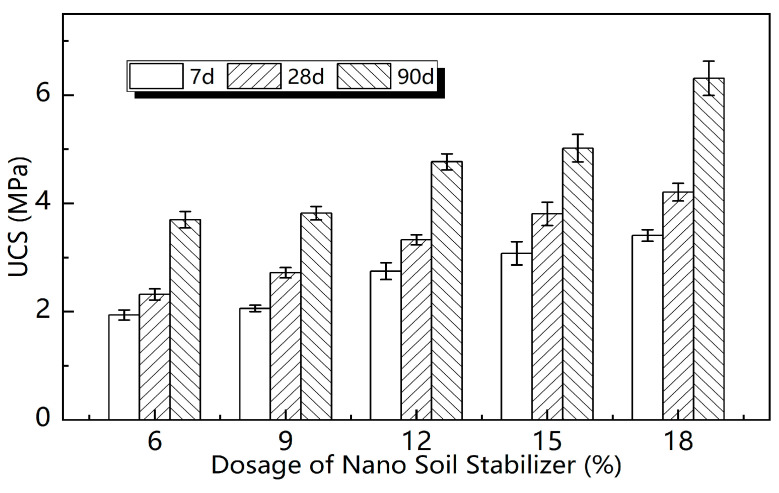
The UCS of nano-stabilized soil by different nano dosages.

Figure 3 presents the unconfined compressive strength of NSS samples at different curing periods of 7 days, 28 days and 90 days. The dosages of N-MBER in NSS were 6%, 9%, 12%, 15% and 18%, respectively. As can be seen from Figure 3, the compressive strength of NSS was related to N-MBER dosage and curing period. On one hand, the UCS of NSS enhanced with the increase in N-MBER dosage, but the growth rate showed a fluctuation. The growth rate of compressive strength gradually increased to around 12%, then decreased, and finally increased again to around 18%. This indicates that the UCS of NSS does not simply increase linearly with the increase in dosage. On the other hand, the compressive strength of the samples developed with the increase in curing period. Compared with 28 days, the compressive strength of the samples at 7 days could reach 75~80% of that at 28 days, and that at 90 days was about 1.3~1.6 times that at 28 days. This result shows that NSS reached a high compressive strength in the early stage, while the UCS still possessed a large growth potential in the later stages of curing, when the conditions were suitable.

**Figure 4:**
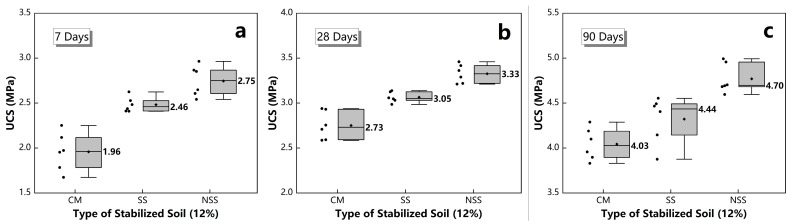
UCS of different stabilized soil under 12% dosage. (**a**) the UCS after 7 days curing; (**b**) the UCS after 28 days curing; (**c**) the UCS after 90 days curing.

According to the above analysis results, the UCS growth rate of the sample was relatively high near the 12% dosage of N-MBER. This indicated that there may be an “optimal dosage” of N-MBER, around which both the intensity and growth rate could reach a peak. It should be noted that the UCS growth rate increased again when the N-MBER dosage increased from 15% to 18%. The reason may be that the silica in NSS converted to the free state ${\mathrm{SiO}}_{3}^{2-}$, which increased the number of anions involved in the hydration reaction and improved the adsorption capacity of Ca^2+^ in the soil colloid. Thus, the secondary hydration reaction of tricalcium silicate and tricalcium aluminate was accelerated to generate more C-S-H gel. However, excessive N-MBER will also produce adverse effects. For example, too many nanoparticles will encase soil particles and slow down the hydration process. In addition, the agglomeration effect of nanoparticles will also reduce their nucleation sites, which is not conducive to the generation of hydration products. In view of this, if the needs of the economy and sustainability are taken into account, this study is more inclined to recommend a 12% dosage of N-MBER for future research and application. In order to demonstrate the strength superiority of NSS, the compressive strengths of three different stabilized soils under a 12% dosage were compared. The results are shown in Figure 4, where the box represents the interquartile range of the data of each group, the bold line is the median line, and whisker indicates the lowest and highest values within the interquartile range 1.5 times from the box. It can be seen in Figure 4 that the UCS of NSS was the highest among the three at each period under the dosage of 12%, at 2.75 MPa, 3.33 MPa and 4.70 MPa, respectively, and about 10–15% higher than SS and 20–40% than CM. The comparison results indicate that N-MBER could improve the mechanical properties of loess more significantly, and that the consolidation effect was better than that of the ordinary stabilizer and 42.5 cement.

The mechanism of the nano-stabilizer improving the uniaxial compressive strength of soil could be divided into the following aspects: firstly, from the aspect of accelerating the hydration process of NSS, the nano SiO_2_ in the N-MBER had high pozzolanic activity [30], which could adsorb more free water on the surface of soil particles [31], accelerate the early hydration process [32] and promote the generation of more hydration products, such as ettringite. Second, the nano SiO_2_ could participate in the hydration of tricalcium silicate, effectively refining the calcium hydroxide (CH) crystals which are unfavorable to strength growth in soil colloid [2], generating a large number of calcium silicate hydrate (C-S-H) gels of different shapes and intertwining with hexagonal prismatic ettringite (Aft) to form a solid three-dimensional spatial network structure [33]. Meanwhile, the N-MBER possessed comparatively obvious superiorities in particle size and specific surface area [12], which could not only effectively fill the micropores inside the soil, but also enhance the van der Waals force and surface energy between molecules, consequently improving the aggregation effect between soil particles significantly [34]. Moreover, N-MBER could be used as the nucleation site of C-S-H to induce the hydration process and make the NSS hydration reaction more sufficient [35,36]. Lastly, the nano-stabilizer could also activate the phase interface of inert minerals and change their filling and cementation forms in soil [37,38]. Consequently, the microstructure of NSS was improved, and the compressive strength and macroscopic properties at each period were significantly enhanced.

### 3.2. Uniaxial Compression Test Results

**Figure 5:**
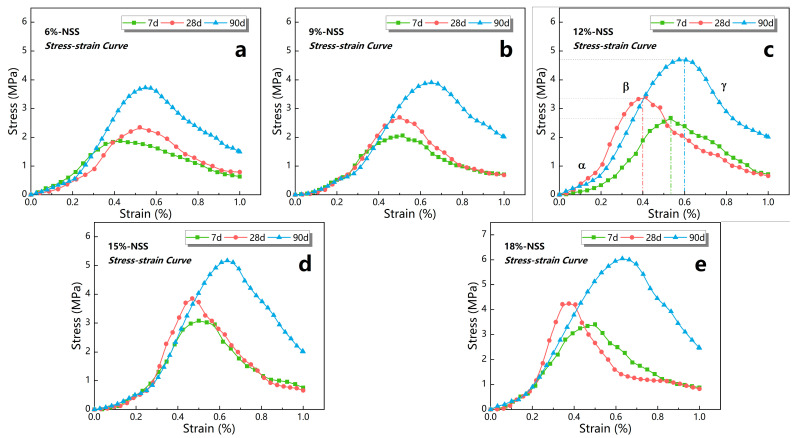
Stress–strain curves of NSS under different dosage at different curing periods. (**a**) Stress–strain curves of NSS under a 6% dosage; (**b**) stress–strain curves of NSS under a 9% dosage; (**c**) stress–strain curves of NSS under a 12% dosage; (**d**) stress–strain curves of NSS under a 15% dosage; (**e**) stress–strain curves of NSS under a 18% dosage.

The failure process of nano-stabilized soil under uniaxial compression is one of significant parameters used to investigate the physical and mechanical properties and stress–strain constitutive model of cement-based stabilized soil [24,39]. In order to clarify this process, the stress–strain data of NSS under different curing periods and dosages were analyzed and summarized in this research, and the results are shown in Figure 5.

The uniaxial compression process of NSS could be divided into two stages, pre-fracture and post-fracture, according to the compressive fracture pattern of the samples. The stress–strain curve showed obvious ascending and descending stages, respectively, which indicated a typical hyperbolic form [23,40]. As seen from Figure 5, first of all, when the dosage of nano-stabilizer increased from 6% to 18%, the peak stress of NSS at each dosage gradually increased, which indicated that the compressive strength was positively correlated with the dosage. Secondly, the peak value of the stress–strain curve of NSS was mainly located between 30 and 70% of the strain, and the slope rate of the curve before the peak of each period increased with the increase in the N-MBER dosage, which indicated that the brittleness of the NSS was gradually reinforced [14,41]. Finally, it was found that the slope of the curve before stress peak increased gradually with the increase in the curing period when the N-MBER dosage was less than 12%, However, it increased first and then decreased with the curing period when the dosage was over 12%. The above variation reveals that the yield strength of NSS reached the maximum when the dosage was near 12%; nevertheless, the strain under the same stress increased progressively when the dosage exceeded 12%, which demonstrated that the compressive properties of the material reduced gradually.

The failure process of NSS under uniaxial compression could be roughly divided into three stages, by comparing the variation law of stress–strain curves at different periods and dosages in Figure 5. α represents the pore closure stage inside the material, which was also the elastic deformation stage. In this stage, the cracks inside the sample were gradually compressed and closed, resulting in nonlinear deformation. Meanwhile, all deformations could be recovered after unloading. β is the phase of linear elastic deformation, when the stress–strain curve was approximately a straight line, and the stress reached about 70% of the peak value. At this stage, the deformation could be completely recovered after unloading, and the samples yielded to a certain extent near the stress peak. γ signifies the failure stage of the material. As shown in this stage, the strain increased slowly while the stress decreased rapidly, and the cracks gradually increased to penetration. Afterwards, obvious plastic deformation occurred, and the sample was broken and unrecoverable.

The above stages summarize the failure process and the microstructure variation of NSS samples under the UCS test, which revealed the difference between nano-stabilized soil and traditional concrete materials in stress and deformation, and provided basic support for the quantitative demonstration of the stress–strain constitutive model at different stages of the uniaxial compression process.

### 3.3. Constitutive Modeling of Uniaxial Compression

#### 3.3.1. Equation Deduction of Constitutive Model

It can be seen in the strength variation and failure process that the NSS had obvious linear-elastic deformation and elastic-plastic deformation under uniaxial compression. Furthermore, the stress–strain curve is usually divided into ascending and descending stages in the investigation of cement-based constitutive models. As a consequence, the stress–strain curve of NSS in this research was also divided into ascending and descending stages to analyze separately, by referring to the methods commonly used in the study of concrete stress–strain constitutive models [23,42].

In order to eliminate the individual differences of NSS caused by human factors in the process of sample preparation, and facilitate the extension and verification of the constitutive model, the stress–strain data of NSS sample were processed without dimensionality, as shown in Equation (1).

(1) $$x=\frac{\varepsilon }{\varepsilon_{p}},\;y=\frac{\sigma }{\sigma_{p}}$$

where $\sigma_{p}$ is the peak stress and σ represents the stress at any point, which are both measured in MPa; $\varepsilon_{p}$ is the strain corresponding to the peak stress and ε represents the strain at any point, both measured in %.

**Figure 6:**
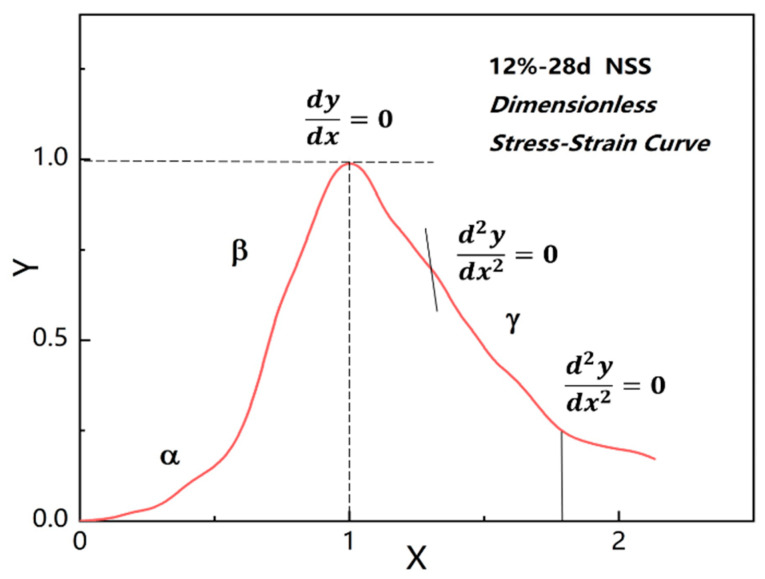
Stress–strain curves after dimensionless processing.

As an example, we analyzed the stress–strain data of the NSS sample cured for a 28-day period under a 12% dosage, and the stress–strain curve after dimensionless processing is shown in Figure 6, where X and Y values were calculated, respectively, according to Equation (1).

It can be found in Figure 6 that the ascending section of the curve possessed the following geometric characteristics: (a) 0≤x≤1 and 0≤y≤1; (b) y=0 when x=0; (c) y=1 and $\frac{dy}{dx}=0$ when x=1, where the curve had a single peak. According to these characteristics, the ascending curve could be formulated by a cubic polynomial [43], as shown in Equation (2).

(2) $$y=ax+\left(3-2a\right)x^{2}+\left(a-2\right)x^{3}$$

where a is the parameter.

The descending stage of the stress–strain curve possesses the following geometric characteristics: (a) y=1 and $\frac{dy}{dx}=0$ when x=1; (b) 0≤y≤1 while $\frac{dy}{dx}<0$, when x≥1; (c) the descent curve has an inflection point at the position where $\frac{d^{2}y}{dx^{2}}=0$, and x>1; (d) the maximum curvature of the descending curve occurs at the point where $\frac{d^{3}y}{dx^{3}}=0$. Based on the above conditions, Equation (3) could be used to simulate the descending stage curve.

(3) $$y=\frac{x}{b{\left(x-1\right)}^{2}+x}$$

where b is the parameter.

According to Equation (3), the curve would pass through the peak point and extend horizontally when b=0 and y=1. At this time, the strain characteristics of the material were plastic deformation under ideal conditions. In addition, the residual strength of the material was 0 after the peak stress, when b→∞ and y→0—in other words, it presented as a completely brittle material [42].

The above simulation and analysis of the ascending and descending stages of the stress–strain curve of NSS were mainly based on the relevant modeling methods in the Code for the Design of Concrete Structures (GB50010-2010) [26,43]. As the basic index of strength characterization, the compression failure process and deformation law of cement-based stabilized soil were also among the main factors affecting the quality of engineering. For this reason, the key to revealing the stress–strain law of NSS under uniaxial compression was to simulate the ascending stage of the curve, while the descending stage of the curve could be analyzed according to the equation of the concrete stress–strain constitutive model.

There existed obvious differences between NSS and concrete in the ascending stage of the stress–strain curve, which were mainly due to the pore closure process in the α stage of the former. In this process, the internal micropores of the sample closed rapidly when compressed; moreover, the stress increased slowly, while the strain varied greatly. In addition, due to the plastic slip of soil particles in the uniaxial compression process, certain plastic yield characteristics near the stress peak in the β stage were observed. In other words, the NSS stress–strain curve roughly demonstrated geometric characteristics of an “S” shape in the ascending stage before the NSS arrived at the peak stress, which showed high geometric similarity with a “logistic” curve from the perspective of the variation trend. Accordingly, combined with the research results of the strength growth model of N-MBER, the “S-Logistic” growth function model could be used to simulate the ascending stage of the NSS stress–strain curve [3,40], where the stress increased slowly and showed an exponential trend in the initial stage, then increased rapidly within a certain range, and finally the growth rate gradually decreased again. The function model is shown in Equation (4).

(4) $$y=\frac{\mu }{1+e^{-v\cdot \left(x-\sigma \right)}}\;$$

where μ, ν, and σ are model parameters, and all are greater than 0.

#### 3.3.2. Parametric Analysis of Constitutive Model

The stress–strain curves of NSS samples under different dosages and curing periods were nonlinearly fitted according to the UCS test results. The parameters of the above constitutive models were calculated based on the interpolation principle of the least square method. The descending stage of the curve was simulated based on the function model of Equation (3) (Con-2010), while the ascending curve was calculated by Equations (2) and (4), respectively. The model parameters are shown in Table 3.

As shown in Table 3, the value of parameter a calculated according to the concrete constitutive model Con-2010 ranged from −1.9 to −0.1, and that of parameter b ranged from 1.9 to 7.3. The negative value of parameter a indicated that the sample was subjected to tensile stress at the beginning of axial compression when using the concrete constitutive model to simulate the curve of NSS ascending stage, which obviously did not conform to reality. It also means that there will exist large errors if the concrete constitutive model is directly used to simulate the curve of the NSS ascending stage. On the other side, the parameter ν calculated by the S-Logistic model was between 6.1 and 7.9 and the parameter σ was between 0.55 and 0.75. In addition, the simulated parameter μ first increased and then decreased with the increase in the curing period, except for the samples under a 12% dosage. It could be found by comparing the simulation results that the ascending stage of the curve calculated by the S-Logistic model was more consistent with the actual law, and the parameters’ distribution range was relatively uniform.

The parameter b of the descending stage simulated by the Con-2010 model basically showed a trend of increasing with the increase in N-MBER dosage. This indicated that the more the N-MBER dosage increased, the steeper the descending stage curve of NSS after the peak stress was. More precisely, the stress decay rate of the sample will be faster, and the material will be more brittle. This also means that the Con-2010 model was suitable for the simulation of the curve descending stage and could achieve relatively accurate results. Compared with other dosages, the value of parameter b with a 12% dosage was slightly reduced, which demonstrated that the NSS sample with a 12% dosage demonstrated higher compressive strength and better ductility, simultaneously. It was verified again from the perspective of the constitutive model that there exists an optimal dosage about 12% of N-MBER in the NSS.

The accuracy of the curves simulated by the above two functional models was compared for the purposes of accurately predicting and formulating the stress–strain constitutive relationship of NSS. The visual fitting tool of Origin software (Version 2019b) was used to analyze the fitting degree of the two models, and the determination coefficient R^2^ and the residual standard deviation RCS were calculated, respectively. The larger value of the R^2^ and the smaller value of the RCS represented the higher accuracy of the model simulation. The parameter fitting results of the two constitutive models are shown in Table 4, where AS indicates the ascending stage and DS represents the descending stage.

According to the comprehensive analysis of the parameter fitting results in Table 3 and Table 4, it can be seen that the determination coefficient R^2^ of the S-Logistic model was larger than that of the Con-2010 model, while the residual standard deviation RCS was also smaller. This indicated that the S-Logistic model could be more accurate in simulating the stress–strain law of NSS under uniaxial compression in the curve’s ascending stage. Therefore, the uniaxial compression stress–strain constitutive model of the NSS constructed in this research is formulated as Equation (5).

(5) $$\{\begin{array}{c} y=\frac{\mu }{1+e^{-v\cdot \left(x-\sigma \right)}}\;,\;\;\;\left(0\leq x\leq 1\right)\\ y=\frac{x}{b{\left(x-1\right)}^{2}+x}\;\;,\;\;\;\;\;\;(x>1) \end{array}$$

where μ, ν, σ, and b are all parameters and are greater than 0.

Compared with the traditional stress–strain constitutive model of cement-based materials, the model established in Equation (5) shows better superiority in describing the linear-elastic and elastic-plastic variation characteristics of NSS under uniaxial compression. Since the stress–strain curve of the NSS was divided into ascending and descending stages, and it was optimized and simulated in different stages; consequently, Equation (5) was not only more explicit in expression form than the previous models, but also more consistent with the actual deformation law of NSS under uniaxial compression in a physical sense. In a sense, the result of this study is also an important supplement to the theoretical research of the stress–strain constitutive model of cement-based stabilized soil.

#### 3.3.3. Experimental Verification of Constitutive Model

**Figure 7:**
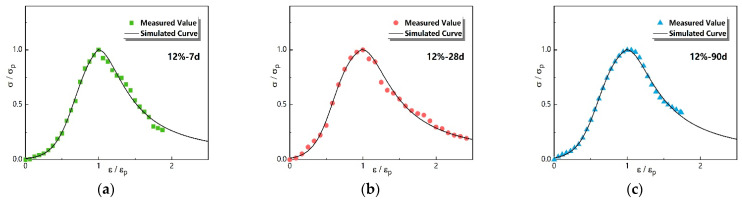
The verification results of the constitutive model. (**a**) verification results of NSS after a 7-day curing period; (**b**) verification results of NSS after a 28-day curing period; (**c**) verification results of NSS after a 90-day curing period.

The rest of the UCS data, under a 12% dosage and which did not participate in model construction, were analyzed and used to verify the constitutive model of Equation (5). The verification results are shown in Figure 7. It can be seen from Figure 7 that the simulated value of the uniaxial compression constitutive model under each curing period possessed a good consistency with the measured value when the dosage of N-MBER was near the optimal one. Meanwhile, the fitting degree of the ascending stage was better than that of the descending one, which indicated that the S-Logistic model was more appropriate than the concrete constitutive model in simulating the stress–strain process of NSS before uniaxial compression failure. Moreover, the simulated curve for the curing period of 28 days was more consistent with the measured values than that for 7 days, while the model fitting accuracy for 90 days achieved the best accuracy among the three groups, which indicated that the simulation accuracy of the model presented an increasing trend with the increase in the curing period.

## 4. Conclusions

The main conclusions of this study include the following points:

(1) The influence factors of nano-stabilized soil under uniaxial compression were analyzed by laboratory typical tests. The results show that the improvement effect of N-MBER on the compressive strength of NSS was positively correlated with the curing period and its dosage, and proposed the optimum dosage of N-MBER to be 12%. The UCS of 12% NSS is more than 10% higher than that of SS, and 25% higher than that of grade 42.5 cm.

(2) Combined with the curve characteristics of the sample compression process, we revealed the different stages of compression deformation of NSS. It was found that there existed different characteristics from concrete in the compression deformation process of NSS, which were mainly manifested in the pore closure stage at the initial compression and the plastic yield stage before the peak stress.

(3) The stress–strain constitutive model of NSS under uniaxial compression was established and verified. It demonstrated that the established constitutive model of NSS under uniaxial compression was more consistent with the material’s actual deformation characteristics, which could simulate the stress–strain curve law precisely after verification.

(4) There are still some unsolved problems in this study. For example, the freeze–thaw cycle characteristics and permeability of NSS have not been revealed, and the stress–strain law of NSS under anisotropic loads also needs to be further studied.

## Figures and Tables

**Table 1:** Composition and content of N-MBER.

Ingredients	Types	Content %
Nanoparticles	Nano SiO_2_	1~3
Gelling Agent	Cement Clinker	75~85
Alkaline Catalyst	Fly Ash	8~10
Grinding Aid	Slag	2~3
Retarder	Gypsum	3~5
Surfactant	Active Agent	1~2

**Table 2:** Mix proportions of each experimental group.

Test Groups	Dosage (%)	Curing Period (Days)		
		7	28	90
NSS	6	A1-1	A1-2	A1-3
	9	A2-1	A2-2	A2-3
	12	A3-1	A3-2	A3-3
	15	A4-1	A4-2	A4-3
	18	A5-1	A5-2	A5-3
SS	12	A0-1	A0-2	A0-3
CM	12	AA-1	AA-2	AA-3

**Table 3:** The constitutive model parameters calculated by the two methods.

Dosage %	Period Days	Con-2010		S-Logistic		
		a	b	μ	ν	σ
6	7	−0.138	1.988	1.085	6.150	0.553
	28	−1.012	4.985	1.113	6.476	0.649
	90	−0.980	4.138	1.097	6.913	0.632
9	7	−0.515	5.511	1.043	7.432	0.561
	28	−1.334	6.784	1.129	6.797	0.680
	90	−1.071	5.626	1.104	7.111	0.641
12	7	−1.348	5.556	1.119	6.932	0.679
	28	−0.729	4.916	1.070	7.331	0.594
	90	−0.776	4.856	1.071	6.905	0.602
15	7	−1.376	6.466	1.149	6.900	0.690
	28	−1.906	7.159	1.148	7.930	0.743
	90	−1.297	7.209	1.092	7.407	0.659
18	7	−0.474	7.295	1.051	7.339	0.560
	28	−1.721	5.914	1.146	7.952	0.717
	90	−0.567	6.173	1.047	6.894	0.572

**Table 4:** The fitting degree of parameters calculated by the two constitutive model.

Dosage %	Period Days	Adj. R^2^			RCS		
		Con-2010		S-Logistic	Con-2010		S-Logistic
		AS	DS	AS	AS	DS	AS
6	7	0.9929	0.9673	0.9969	0.0010	0.0018	0.0005
	28	0.9663	0.9953	0.9958	0.0047	0.0003	0.0007
	90	0.9784	0.9934	0.9992	0.0030	0.0003	0.0001
9	7	0.9932	0.9742	0.9964	0.0010	0.0013	0.0006
	28	0.9592	0.9952	0.9978	0.0057	0.0003	0.0004
	90	0.9762	0.9836	0.9984	0.0033	0.0005	0.0002
12	7	0.9514	0.9756	0.9982	0.0065	0.0015	0.0003
	28	0.9869	0.9777	0.9970	0.0020	0.0014	0.0006
	90	0.9885	0.9862	0.9996	0.0016	0.0007	0.0001
15	7	0.9530	0.9901	0.9969	0.0067	0.0007	0.0005
	28	0.8723	0.9902	0.9971	0.0170	0.0008	0.0004
	90	0.9636	0.9932	0.9993	0.0049	0.0003	0.0001
18	7	0.9947	0.9676	0.9977	0.0008	0.0019	0.0004
	28	0.9198	0.9591	0.9963	0.0119	0.0027	0.0007
	90	0.9962	0.9945	0.9991	0.0005	0.0003	0.0001

## Data Availability

Not applicable.
